# Carbon nanotube superplastic with long-awaited performance

**DOI:** 10.1093/nsr/nwag271

**Published:** 2026-05-11

**Authors:** Rong Xiang, Shigeo Maruyama

**Affiliations:** School of Mechanical Engineering, Zhejiang University, China; School of Mechanical Engineering, Zhejiang University, China; Department of Mechanical Engineering, The University of Tokyo, Japan; Institutes of Innovation for Future Society, Nagoya University, Japan

Plastics are widely used in modern daily living. They are light-weight and easy to fabricate into different shapes, but are usually not good conductors of heat and electricity, which has limited their application in many fields. One way of altering the properties of a plastic involves the incorporation of an additive phase. For example, introducing carbon nanotubes (CNTs) can improve the thermal and electric conductivity of a polymer. However, conventional methods usually need harsh pretreatments, which deteriorates the intrinsic high conductance of the materials. As a result, the properties of these obtained CNT–polymer composites are usually way less than the expectation.

**Figure 1.**
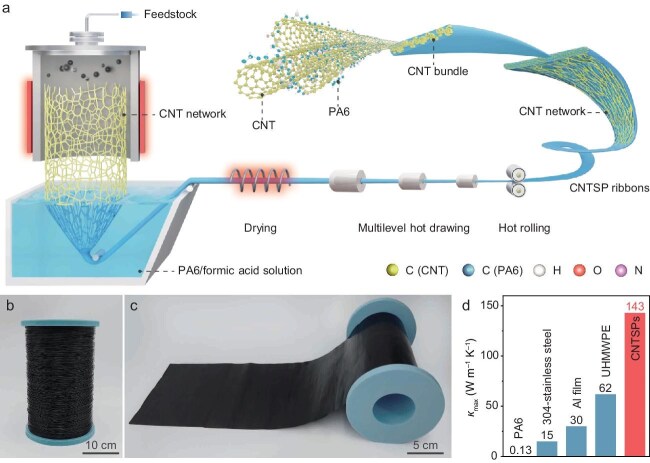
(a) Fabrication flow of CNT-based superplastics. (b and c) Pictures of hundreds of meters of long fibers and tens of centimeters wide ribbons; (d) thermal conductivity of CNT superplastics showing an over 1000-time improvement from the initial PA6. Reproduced from Ref. [1] with permission.

However, in a recent report, Li and co-workers have achieved a CNT-enhanced plastic with long-awaited superior performances [1]. The material holds a high thermal conductivity of 143 ± 5.8 W m^−1^ K^−1^, an over three orders of magnitude improvement from the original PA6 plastic. It also exhibits a remarkable tensile strength of 663 ± 18 MPa, stronger than common Al alloy. Also, the composite has an electrical conductivity of 8.6 × 10^4^ S m^−1^, meaning the polymer is already as conductive as some metals. This high electric conductivity, together with its fast thermal transport, makes it useful not only in antistatic, but maybe directly as a heating unit. Most importantly, these CNT–polymers are already available as hundreds of meters long fibers and tens of centimeters wide ribbons (Fig. 1).

Two technologies are innovated here and are responsible for the outstanding properties of the final plastic. First, the authors have employed an *in situ* mixing method. Well crystallized, aligned, long CNTs are prepared by a floating catalytic process [2] and collected directly from the hot furnace and spun into a fiber together with PA6 or formic acid solution. This *in situ* process avoids conventional pretreatments such as dispersion that inevitably brings defects into the atomic skeleton of CNTs. Benefiting from this damage-free processing, the intrinsic structural integrity at the atomic scale and the high degree of alignment in macroscopic scale are both preserved throughout the whole fabrication.

Second, the authors have utilized a home-developed multilevel hot drawing and rolling process. Initially prepared fibers contain solvent and unnecessary pores. A sophisticated and optimized post-treatment is therefore critical for the final geometry and properties of the composite. Here, continuous drawing will not only improve the alignment, but also the non-uniform initial strain inside a CNT fiber [3]. Hot rolling will allow the densification of the structures in order to shape them into ribbons, which are then convenient for further manufacturing.

One striking feature of this superplastic is that it reaches a thermal conductivity anisotropy ratio of ∼123, meaning heat can conduct over 100 times faster in one direction (along the CNT) than directions perpendicular to the CNT axis. This anisotropy, together with the high processibility of the plastic, allows the CNT superplastic to be molded into different shapes and designed functions using existing polymer manufacturing techniques. A 3D-printed heat sink is presented as an example, and one can imagine more.

In summary, Li and co-workers have demonstrated important progress for CNT–polymer composites, where properties of individual CNTs are preserved and transferred into macroscopic products. Therefore, these flexible, processible, mass-producible fibers and ribbons with long-awaited good properties could serve as the starting unit for many commercial products and open a new era of CNT-based super polymers [4].
